# First evaluation of a commercial multiplex PCR panel for rapid detection of pathogens associated with acute joint infections

**DOI:** 10.5194/jbji-8-45-2023

**Published:** 2023-01-18

**Authors:** Jorrit Willem Adriaan Schoenmakers, Rosanne de Boer, Lilli Gard, Greetje Anna Kampinga, Marleen van Oosten, Jan Maarten van Dijl, Paulus Christiaan Jutte, Marjan Wouthuyzen-Bakker

**Affiliations:** 1 Department of Orthopaedic Surgery, University of Groningen, University Medical Center Groningen (UMCG), Groningen, The Netherlands; 2 Department of Medical Microbiology and Infection Prevention, University of Groningen, UMCG, Groningen, The Netherlands

## Abstract

**Background**: prompt recognition and identification of the causative microorganism in acute septic arthritis of native and prosthetic joints is vital to increase the chances of successful treatment. The aim of this study was to independently assess the diagnostic accuracy of the multiplex BIOFIRE^®^ Joint Infection (JI) Panel (investigational use only) in synovial fluid for rapid diagnosis. **Methods**: synovial fluid samples were collected at the University Medical Center Groningen from patients who had a clinical suspicion of a native septic arthritis, early acute (post-operative, within 3 months after arthroplasty) periprosthetic joint infection (PJI) or late acute (hematogenous, ≥3 months after arthroplasty) PJI. JI Panel results were compared to infection according to Musculoskeletal Infection Society criteria and culture-based methods as reference standard. **Results**: a total of 45 samples were analysed. The BIOFIRE JI Panel showed a high specificity (100 %, 95 % confidence interval (CI): 78–100) in all patient categories. Sensitivity was 83 % (95 % CI: 44–97) for patients with a clinical suspicion of native septic arthritis ( n=12 ), 73 % (95 % CI: 48–89) for patients with a clinical suspicion of a late acute PJI ( n=14 ), and 30 % (95 % CI: 11–60) for patients with a clinical suspicion of an early acute PJI ( n=19 ). **Conclusion**: the results of this study indicate a clear clinical benefit of the BIOFIRE JI Panel in patients with a suspected native septic arthritis and late acute (hematogenous) PJI, but a low clinical benefit in patients with an early acute (post-operative) PJI due to the absence of certain relevant microorganisms, such as *Staphylococcus epidermidis*, from the panel.

## 1 Introduction

Acute bacterial infections in native and prosthetic joints can be a true musculoskeletal emergency in orthopaedic surgery. In septic arthritis of native joints, joint destruction rapidly occurs when not treated timely (Mathews et al., 2010). In addition, in acute periprosthetic joint infections (PJIs), ongoing biofilm formation results in bacterial recalcitrance to antimicrobial therapy and reduces the chance of infection control with retention of the implant (Rosman et al., 2021; Löwik et al., 2020; Wouthuyzen-Bakker et al., 2019a). Therefore, prompt recognition and identification of the causative microorganisms and their susceptibility towards antimicrobials are essential for treatment success. The current standard for detection and identification of the causative organism is based on culturing of synovial fluid and/or interarticular tissue (Arvieux and Common, 2019; Hassan et al., 2017), but this approach has several disadvantages (Trebse and Roskar, 2021; Gottlieb et al., 2019; Fye, 2008): (i) culture results typically take hours to several days which makes this method unsuitable as a rapid diagnostic tool; (ii) culture results can be false negative due to the administration of antibiotic therapy; and (iii) certain bacterial species can be difficult to cultivate, especially in the context of a biofilm associated infection.

The polymerase chain reaction (PCR), an enzymatic assay where bacterial DNA is amplified and labelled with a specific stain for detection, has become increasingly recognized as an elegant tool for identification of specific pathogens or genes due to faster result in comparison to culturing and the feasibility to identify non-dividing or difficult to culture pathogens (Garibyan and Avashia, 2013). This assay does, however, require specific expertise and equipment. The present study was therefore aimed at investigating the diagnostic accuracy of the BIOFIRE^®^ Joint Infection (JI) Panel, which is a fully automated syndromic multiplex PCR panel designed to identify 31 clinically relevant pathogens and eight antimicrobial resistance genes directly from synovial fluid in approximately 1 h.

## 2 Methods

### 2.1 Clinical specimens

Synovial fluid samples were prospectively collected at the University Medical Center Groningen (UMCG) between January 2016 and December 2022 from patients who had a clinical suspicion of a native septic arthritis, early acute (postoperative) PJI (i.e. a sudden onset of an acute warm, painful, and swollen prosthetic joint within 3 months after arthroplasty) or late acute (hematogenous) PJI (i.e. the abovementioned symptoms ≥3 months after arthroplasty). PJIs were diagnosed according to the criteria proposed by the 2013 Musculoskeletal Infection Society (MSIS) (Parvizi and Gehrke, 2014), and septic arthritis according to a positive synovial fluid and/or tissue culture. Exclusion criteria were insufficient volume of synovial fluid for the BIOFIRE assay (200 µL ). The samples were either prospectively or retrospectively analysed. The retrospective samples were collected from an already existing synovial fluid biobank in the UMCG, in which synovial fluid samples of patients with a clinical suspicion of septic arthritis have consecutively been collected and stored at - 80 ${}^{\circ }$ C until analysis. All collected samples were obtained under sterile conditions either by arthrocentesis or intraoperatively. If surgical debridement was performed, an average of five intraarticular biopsies were collected for culture. If mobile parts of the prosthesis were exchanged, these components were sonicated and sonication fluid was cultured. All samples were routinely analysed by standard microbiological work-up according to the protocol previously described by Talsma et al. (2021). For the identification of cultured pathogens, matrix-assisted laser desorption ionization–time of flight mass spectroscopy (MALDI-TOF, Bruker Microflex MS) was used. Furthermore, streptococci were classified according to Lancefield grouping. For the BIOFIRE JI assay, synovial fluid samples were analysed according to the manufacturer's instructions (see below).

### 2.2 The BIOFIRE Joint Infection Panel (investigational use only)

The BIOFIRE JI Panel (BioFire^®^, bioMérieux, Salt Lake City, United States) is an IUO (investigational use only; currently not approved for clinical diagnostic purposes) multiplex assay aimed at simultaneously detecting 31 Gram-positive bacteria, Gram-negative bacteria, and yeast known to be associated with bone and joint infections. In addition, eight antimicrobial resistance genes can be detected by this assay. The microorganisms and antimicrobial resistance genes present in the BIOFIRE JI Panel can be found in Supplement Table S1. For the analysis, 200 µL of synovial fluid was added to the BIOFIRE JI sample buffer, injected into the test pouch, and loaded into the FilmArray^®^ Torch System. Subsequently, an automated assay lysed the cells by using beads, nucleic acids were purified by wash buffer, PCR amplification occurred by primer pairs, and finally detection of the targeted pathogens and resistance genes occurred by using fluorescent binding dyes. The FilmArray software then confirmed the presence or absence of each microorganism or resistance gene within approximately 60 min after loading the sample.

**Table 1:** Characteristics of patients with a clinical suspicion of native septic arthritis, early, or late acute PJIs.

Patient characteristics	Native septic arthritis ( n=12 )	Early acute PJI ( n=14 )	Late acute PJI ( n=19 )
Age (mean and standard deviation)	55±25	67±10	64±15
Joint involved (percentage of total)			
Knee	66.7	21.4	63.2
Hip	25.0	64.3	36.8
Shoulder	8.3	14.3	–
Collection method (percentage of total)			
Arthrocentesis	75.0	28.6	89.5
Surgical	25.0	71.4	10.5

### 2.3 Statistical analyses

Separate analyses were performed for “on-panel” microorganisms (i.e. the 31 microorganisms present in the BIOFIRE JI Panel) and all microorganisms (i.e. microorganisms that are not present in the JI Panel in addition to the “on-panel” microorganisms). Sensitivity (on-panel) for PJIs was calculated as the percentage of correct BIOFIRE PCR results among patients with a PJI according to MSIS criteria who presented a corresponding “on-panel” microorganism in a synovial fluid and/or ( ≥2 ) intra-operative culture. Sensitivity for all microorganisms was calculated as the percentage of correct BIOFIRE PCR results among patients with a PJI according to MSIS criteria who presented any of the corresponding microorganisms in a synovial fluid and/or ( ≥2 ) intra-operative culture. Specificity was calculated as the percentage of negative PCR results among samples diagnosed as not infected according to MSIS criteria. For septic arthritis, the “on panel” sensitivity was calculated as the percentage of positive PCR results among patients presenting an identical “on-panel” microorganism in a synovial fluid and/or ( ≥2 ) intra-operative culture, whereas for all microorganism sensitivity, this could be any microorganism in a synovial fluid and/or ( ≥2 ) intra-operative culture. The specificity was calculated as the percentage of negative PCR results among samples diagnosed as not infected (no positive cultures). Binomial 95 % confidence intervals (CIs) were calculated according to the Wilson method.

## 3 Results

A total of 51 synovial fluid samples (derived from 51 patients, one sample per patient) were collected during the study period. Six collected samples were excluded because of insufficient volume (200 µL ) for the BIOFIRE JI Panel assay. Of the samples, 19 were prospectively analysed (onward from 1 July 2021), and 26 were retrospectively analysed.

### 3.1 Patient characteristics

Of the 45 included samples, 12 were derived from patients with a clinical suspicion of an acute septic arthritis of a native joint, 14 from patients with a clinical suspicion of an early acute PJI, and 19 from patients with a clinical suspicion of a late acute PJI (Table 1). Most synovial fluid samples of patients with a suspected early acute PJI were obtained intraoperatively, while most samples from cases with suspected native septic arthritis or late acute PJIs were obtained by arthrocentesis. From the total cohort, 31 patients were diagnosed with an infection (67 %).

### 3.2 Comparison of the BIOFIRE JI Panel with culture results

Table 2 shows the molecular yield of the BIOFIRE JI Panel. Of the synovial fluid samples, 22 tested positive (49 %), including 10 different bacterial species. No samples were polymicrobial and no antimicrobial resistance genes were detected according to the BIOFIRE results.

**Table 2:** Results of the BIOFIRE JI Panel from 45 synovial fluid samples obtained from patients with a clinical suspicion of native septic arthritis, early, or late acute PJIs.

BIOFIRE JI Panel result	Number of results		Native septic arthritis	Early acute PJI	Late acute PJI
Bacteria					
*Staphylococcus aureus*	6		1	1	4
*Staphylococcus lugdunensis*	1		–	–	1
*Streptococcus pyogenes*	2		1	–	1
*Streptococcus pneumoniae*	1		1	–	–
*Streptococcus agalactiae*	1		–	1	–
Other *Streptococcus* species	6		1	1	4
*Enterobacter cloacae* complex	2		–	1	1
*Haemophilus influenzae*	1		1	–	–
*Enterococcus faecalis*	1		–	1	–
*Escherichia coli*	1		–	–	1
No microorganisms detected	23		7	9	7
Antimicrobial resistance genes detected	0		–	–	–

An overview of the results of the BIOFIRE JI Panel in relation to the culture results of each individual patient and the infection diagnosis is shown in Supplement Table S2. When only the microorganisms present in the JI Panel are taken into account (i.e. “on-panel analysis”), the BIOFIRE JI Panel result was accurate in 39 of 45 (86.7 %) samples, with a sensitivity of 80.6 % (95 % CI: 64–91) and a specificity of 100 % (95 % CI: 78–100). In five synovial fluid samples in the “on-panel” analysis, the BIOFIRE JI Panel was false negative (*Streptococcus pyogenes*, *Streptococcus mutans*, *Staphylococcus lugdunensis*, *Enterobacter cloacae*, and *Cutibacterium avidum*), whereas there were no false positive results.

When all microorganisms were included in the analysis (i.e. also including microorganisms not present in the BIOFIRE JI Panel,), the overall diagnostic performance of the BIOFIRE was lower, with a sensitivity of 61.3 %, while the specificity remained 100 % (Table 3). The additional microorganisms not included in the JI Panel that were detected by standard microbiology workup included *Staphylococcus epidermidis*, *Corynebacterium* species, *Staphylococcus capitis*, *Staphylococcus haemolyticus*, and *Campylobacter jejuni* (Supplement Table S2).

In Table 3, the test accuracy for all microorganisms represented in the BIOFIRE JI Panel is shown per patient category. Notably, a good diagnostic performance is seen in the group of patients with a clinical suspicion of native septic arthritis (sensitivity 83 %, specificity 100 %) and in patients with a clinical suspicion of a late acute (hematogenous) PJI (sensitivity 73 %, specificity 100 %), while a low diagnostic accuracy is seen for patients with a clinical suspicion of an early acute (post-operative) PJI (sensitivity 30 %, specificity 100 %).

**Table 3:** Test accuracy of the BIOFIRE JI Panel for infection according to criteria and cultures (Methods section) as reference standard in patients with a clinical suspicion of native septic arthritis, early, or late acute PJIs.

All samples ( n=45 )		95 % CI	Native septic arthritis	95 % CI	Early acute PJI	95 % CI	Late acute PJI	95 % CI
			( n=12 )		( n=14 )		( n=19 )	
% infection diagnosis	6/12 (50 %)		6/12 (50 %)		10/14 (71 %)		15/19 (80 %)	
% BIOFIRE correct	73.3 %		91.7 %		50.0 %		78.9 %	
Sensitivity	61.3 %	44–76	83.3 %	44–97	30.0 %	11–60	73.3 %	48–89
Specificity	100 %	78–100	100 %	61–100	100 %	51–100	100 %	51–100

### 3.3 Comparison of the BIOFIRE JI Panel with Gram staining

Gram staining of synovial fluid was positive in 32 % of patients diagnosed with an infection (10/31; four samples with Gram-positive cocci in chains, four samples with Gram-positive cocci in clusters, one sample with Gram-positive cocci, and one sample with Gram-negative rods). The BIOFIRE JI Panel correctly identified the causative microorganism in nine of these samples. In one sample with Gram-negative rods, the BIOFIRE did not detect the microorganism, while standard microbiological cultures isolated a *C. jejuni*, which is a microorganism not included in the JI Panel. In addition, the BIOFIRE JI Panel correctly identified the causative microorganisms in 12 samples with a negative Gram stain.

## 4 Discussion

The results of this study indicate that the BIOFIRE JI Panel has clinical potential for patients with a suspected acute septic arthritis of native and prosthetic joints that are hematogenous in origin. Rapid detection of the causative microorganism in these cases may allow for immediate tailoring of antibiotic treatment and a positive test may prompt the surgeon for surgical lavage in doubtful clinical cases. The BIOFIRE JI Panel probably has less clinical potential for patients suspected for an early acute (post-operative) PJI, due to its polymicrobial nature and involvement of certain microorganisms that are not present in the Panel (e.g. the second most common pathogen in early acute PJI, *S. epidermidis*, is not in the JI Panel, which is discussed further below).

In recent years, syndromic multiplex PCR has become increasingly recognized as a reliable diagnostic tool for detection of infection due to its quick results and its user friendliness. When comparing our results to other studies, similar results can be observed. Berneking et al. (2022) used a syndromic panel which targeted bone and joint infection in synovial fluid in patients with a suspicion of periprosthetic joint infections, and reported a sensitivity of 85 % and a specificity of 89 %. Lausmann et al. (2017) used multiplex PCR to target acute PJIs in synovial fluid of patients who underwent surgical irrigation and debridement of their prosthetic joint, and found a sensitivity of 86 % and a specificity of 100 %. Mainly, the (near) perfect specificity in our and other studies highlights the reliability of this diagnostic technique when a positive test result is obtained. A negative test result does not fully exclude infection, but it significantly decreases the a priori chance of infection in patients with a suspicion of native septic arthritis and hematogenous PJI. This can aid clinicians in narrowing down empirical antibiotic therapy within hours instead of days after the onset of symptoms, which will not only protect the joint from further damage but also favours correct antibiotic stewardship to prevent antimicrobial resistance (Cunha and Opal, 2018). The superiority of this technique was additionally highlighted by its outperformance of Gram staining, which is hampered by moderate sensitivity and is therefore not implemented in all medical institutions. However, despite this limitation, Gram staining is currently the only conventional diagnostic method available for obtaining rapid results and therefore it is still used in our hospital (Wouthuyzen-Bakker et al., 2019b). Other possible abilities of the BIOFIRE JI Panel, such as detection of culture-negative infection (e.g. when antibiotics have been administered), were not seen in our study but can be useful as shown by Berneking et al. (2022).

The BIOFIRE JI Panel includes many important microorganisms that are clinically associated with hematogenous septic arthritis of native and prosthetic joints, which are *S. aureus* ( ∼ 50 % of cases), streptococci ( ∼ 16 % to 30 %), and most clinically relevant Gram-negative bacteria ( ∼ 15 % to 20 %), such as *Pseudomonas aeruginosa* and *E. coli* (Benito et al., 2019; Ross, 2017). Rarer causative microorganisms of hematogenous septic arthritis, such as *H. influenzae*, are included in the BIOFIRE JI Panel as well, and this bacterium was also successfully identified in one of our samples. However, for patients with a clinical suspicion of early acute PJIs, the BIOFIRE JI Panel seems less useful, because these post-operative infections are often polymicrobial in nature and include microorganisms of the skin, like coagulase-negative staphylococci such as *S. epidermidis*, of which not all species are included in the JI Panel (Benito et al., 2019; Tande and Patel, 2014). In our study, we found 6 of 45 (13 %) cases of polymicrobial infection, of which five cases were early PJIs. In 4 out of 5 (80 %) early acute PJI cases, the BIOFIRE PCR result was inadequate because the causative microorganisms were not present in the panel. Other bacterial species that the BIOFIRE JI Panel did not detect in our study, *Corynebacterium* species and *Campylobacter* species, occur more sporadically and are therefore arguably less clinically relevant. Nevertheless, for our present patient cohort, the (especially negative) results of the BIOFIRE JI Panel must be interpreted with caution due to the absence of certain relevant bacterial species in the JI Panel, such as *S. epidermidis* and some other coagulase-negative species.

A limitation of this pilot study is its relatively small sample size, which resulted in rather wide 95 % confidence intervals in the subanalysis of the three separate patient categories. The samples that were used for this study were considered diagnostic waste after routine microbiological workup had been completed. Consequently, we did not always receive a sufficient volume of synovial fluid for our research. The six exclusions referred to in our study related to samples that we had received for research but of which the volume was insufficient for our studies. Probably even more synovial fluid samples from patients never reached our research laboratory, because they were “excluded” by the diagnostic laboratory as they had been completely used for diagnostic purposes. Thus, the analysed samples represent ∼ 33 % of all infection cases in our hospital, as we treat thrice as many acute PJIs per year. In turn, this may result in some degree of sampling bias. However, the microorganisms detected in this study did represent the species found in native septic arthritis and acute PJIs, but studies with larger sample sizes are needed to validate these results. Moreover, in our study cohort, a higher percentage of patients was diagnosed with infection (67 %) than might be expected based on population prevalence. This is explained by the fact that our samples were collected in an academic hospital where more complex (infection) cases are referred to from other peripheral hospitals, therefore increasing the likelihood of infection in our cohort. This may also result in sampling bias. In our study cohort, there were no culture-negative PJIs, which is explained by the strict work-up in our institution and by the fact that none of the patients were on prior antibiotic treatment, which is the main reason for culture-negative PJIs (Kalbian et al., 2020). Another limitation of the study is that a fraction of the samples was retrospectively analysed. However, all samples were stored at - 80 ${}^{\circ }$ C until analysis, and we did not find any differences in the diagnostic accuracy between retrospectively and prospectively analysed samples (Supplement Table S2).

In conclusion, the results of this pilot study demonstrate the high accuracy of the BIOFIRE JI Panel in patients with a suspected acute septic arthritis of native or prosthetic joints that are hematogenous in origin. These results indicate that the BIOFIRE JI Panel may potentially lead to better antibiotic stewardship and patient treatment in this patient category. Larger studies are needed to confirm these results and to determine to what extent the BIOFIRE JI Panel will potentially alter patient management.

## Supplement

10.5194/jbji-8-45-2023-supplement In Supplement Table S1, an overview of microorganisms and antimicrobial resistance genes present in the BIOFIRE JI Panel are shown. In Supplement Table S2, all BIOFIRE JI Panel and culturing results are shown. The supplement related to this article is available online at: https://doi.org/10.5194/jbji-8-45-2023-supplement.

## Data Availability

The Supplement tables provide all the data which were used for the analyses.
