# Supplementary material for: First evaluation of a commercial multiplex PCR panel for rapid detection of pathogens associated with acute joint infections
Source: J Bone Jt Infect. 2023 Jan 18;8(1):45–50. doi: 10.5194/jbji-8-45-2023 (PMC9901515; doi:10.5194/jbji-8-45-2023)
Supplement: In Supplement Table S1, an overview of microorganisms and antimicrobial resistance genes present in the BIOFIRE JI Panel are shown. In Supplement Table S2, all BIOFIRE JI Panel and culturing results are shown. The supplement related to this article is available online at: https://doi.org/10.5194/jbj [file jbji-8-45-supplement.pdf]

Supplement of J. Bone Joint Infect., 8, 45–50, 2023
https://doi.org/10.5194/jbji-8-45-2023-supplement
© Author(s) 2023. CC BY 4.0 License.

Supplement of

First evaluation of a commercial multiplex PCR panel for rapid detection of pathogens associated with acute joint infections

Jorrit Willem Adriaan Schoenmakers et al.

Correspondence to: Marjan Wouthuyzen-Bakker (m.wouthuyzen-bakker@umcg.nl)

The copyright of individual parts of the supplement might differ from the article licence.

**BIOFIRE Joint Infection Panel Targets**		
**Gram-positive bacteria**	**Gram-negative bacteria**	**Yeasts**
Anaerococcus prevotii/vaginalis	*Bacteroides fragilis*	*Candida spp.***
Clostridium perfringens	*Citrobacter*	*Candida albicans*
Cutibacterium avidum/granulosum	*Enterobacter cloacae complex*	
Enterococcus faecalis	*Escherichia coli*	
Enterococcus faecium	*Haemophilus influenzae*	
Finegoldia magna	*Kingella kingae*	
Parvimonas micra	*Klebsiella aerogenes*	
Peptoniphilus	*Klebsiella pneumoniae group*	
Peptostreptococcus anaerobius	*Morganella morganii*	
Staphylococcus aureus	*Neisseria gonorrhoeae*	
Staphylococcus lugdunensis	*Proteus spp.*	
*Streptococcus spp**	*Pseudomonas aeruginosa*	
Streptococcus agalactiae	*Salmonella spp.*	
Streptococcus pneumoniae	*Serratia marcescens*	
Streptococcus pyogenes		
**Antimicrobial resistance genes**		
Carbapenemases		
IMP, KPC, NDM, Oxa-48-like, VIM		
Methicillin Resistance		
mecA/C and MREJ		
Extended Spectrum Beta-Lactamase		
CTX-M		
Vancomycin Resistance		
vanA/B		

Table S1. Overview of microorganisms and antimicrobial resistance genes present in the BIOFIRE JI Panel. Data can be found online (BIOFIRE JI Panel: https://www.biofiredx.com/products/the-filmarray-panels/ji/, last access: 30 November 2022). * All streptococci are included in the JI Panel, but only *S. agalactiae*, *S. pneumoniae* and *S. pyogenes* results are narrowed down to specific specie. For the other streptococci species, the BIOFIRE JI result is *''Streptococcus spp''*. ** As mentioned before, but now applicable to candida species.

Pt nr	Clinical suspicion of	Infection by criteria?	BIOFIRE JI result	Synovial fluid culture	Tissue cultures	Sonication fluid culture	Gram Staining
1	Acute native	Yes, SA	*S. pneumoniae*	*S. pneumoniae*	-	-	No bacteria or yeast ****
2	Acute native	Yes, SA	*S. pyogenes*	*S. pyogenes*	Negative	-	No bacteria or yeast ****
3	Acute native	Yes, SA	*St. aureus*	*St. aureus*	*St. aureus* (4/5)	-	No bacteria or yeast ****
4	Acute native	Yes, SA	*H. influenzae*	*H. influenzae*	Negative	-	No bacteria or yeast ****
5	Acute native	Yes, SA	*Streptococcus spp*	*S. dysgalactiae/canis*	*S. dysgalactiae/canis* (1/2)	-	Gram-positive cocci
6	Early acute PJI	Yes, PJI	*E. cloacae complex*	*E. cloacae complex*	-	-	Not performed
7	Early acute PJI	Yes, PJI	*St. aureus*	*St. aureus*	*St. aureus* (6/6)	*S. aureus*	No bacteria or yeast ****
8	Early acute PJI	Yes, PJI	*Streptococcus spp* **	*S. mitis* + *St. epidermidis* + *Corynebacterium spp.*	*S. capitis* (5/6) + *St. epidermidis* (6/6) + *Corynebacterium spp.* (6/6) + *S. mitis* (5/6)	-	No bacteria or yeast ****
9	Early acute PJI	Yes, PJI	*S. agalactiae*	*S. agalactiae (group B)*	*S. agalactiae (group B)* (4/4)	*S. agalactiae (group B)*	No bacteria or yeast ****
10	Early acute PJI	Yes, PJI	*En. faecalis* **	*En. faecalis* + *C. tuberculostearicum*	*En. faecalis (5/5)* + *C. tuberculostearicum* (3/5)	-	No bacteria or yeast ****
11	Late acute PJI	Yes, PJI	*Streptococcus spp.*	*S. dysgalactiae*	-	-	Gram positive cocci in chains
12	Late acute PJI	Yes, PJI	*Es. coli*	*Es. coli*	*Es. coli* (2/4)	-	No bacteria or yeast ****
13	Late acute PJI	Yes, PJI	*St. aureus*	*St. aureus*	*St. aureus* (2/4)	Negative	Gram-positive cocci in clusters
14	Late acute PJI	Yes, PJI	*S. pyogenes*	*S. pyogenes (group A)*	*S. pyogenes (group A)* (4/5)	*S. pyogenes (group A)*	Gram positive cocci in chains
15	Late acute PJI	Yes, PJI	*Streptococcus spp.* **	*S. vestibularis*	*S. vestibularis* (5/5) + *St. capitis* (5/5) + *St. epidermidis* (2/5)	-	Gram positive cocci in chains
16	Late acute PJI	Yes, PJI	*St. aureus*	*St. aureus*	-	-	Gram-positive cocci in clusters
17	Late acute PJI	Yes, PJI	*St. lugdunensis*	*St. lugdunensis*	*St. lugdunensis* (5/5)	-	No bacteria or yeast ****
18	Late acute PJI	Yes, PJI	*Streptococcus spp.*	*S. mutans*	*S. mutans* (4/4)	*S. mutans*	No bacteria or yeast ****
19	Late acute PJI	Yes, PJI	*Streptococcus spp.*	*S. dysgalactiae/canis*	*S. dysgalactiae/canis* (3/4)	-	Gram positive cocci in chains
20	Late acute PJI	Yes, PJI	*E. cloacae complex*	*E. cloacae complex*	*E. cloacae complex* (5/5)	*E. cloacae complex*	No bacteria or yeast ****
21	Late acute PJI	Yes, PJI	*St. aureus*	*St. aureus*	*St. aureus* (3/5)	*St. aureus*	Gram-positive cocci in clusters
22	Late acute PJI	Yes, PJI	*St. aureus*	*St. aureus*	-	-	Gram-positive cocci in clusters
23	Acute native	Yes, SA	Negative *	*S. pyogenes (group A)*	*S. pyogenes (group A)* (1/1)	-	No bacteria or yeast
24	Acute native	No	Negative	Negative	-	-	Not performed
25	Acute native	No	Negative	Negative	-	-	No bacteria or yeast
26	Acute native	No	Negative	Negative	Negative	-	No bacteria or yeast
27	Acute native	No	Negative	Negative	Negative	-	No bacteria or yeast
28	Acute native	No	Negative	Negative	Negative	-	No bacteria or yeast
29	Acute native	No	Negative	Negative	Negative	-	No bacteria or yeast
30	Early acute PJI	No	Negative	Negative	Negative	Negative	No bacteria or yeast
31	Early acute PJI	Yes, PJI	Negative *	Negative	*Cu. avidum* (3/3) + *St. epidermidis* (1/3)	-	No bacteria or yeast
32	Early acute PJI	No	Negative	Negative	Negative	Negative	Not performed
33	Early acute PJI	Yes, PJI	Negative *	*E. cloacae complex* +*St. epidermidis*	*E. cloacae complex* (4/4) + *St. epidermidis* (3/4)	*E. cloacae complex*	No bacteria or yeast
34	Early acute PJI	Yes, PJI	Negative **	Negative	*St. epidermidis* (3/5) + *C. aurimucosum* (2/5) + *St. caprae* (1/5)	*St. epidermidis + St. haemolyticus*	No bacteria or yeast
35	Early acute PJI	No	Negative	Negative	Negative	Negative	No bacteria or yeast
36	Early acute PJI	Yes, PJI	Negative **	*St. epidermidis*	*St. epidermidis* (5/5)	*St. epidermidis*	No bacteria or yeast
37	Early acute PJI	Yes, PJI	Negative **	*St. capitis*	*St. capitis* (1/5)	*St. capitis*	No bacteria or yeast
38	Early acute PJI	No	Negative	Negative	Negative	Negative	No bacteria or yeast
39	Late acute PJI	No	Negative	Negative	Negative	-	No bacteria or yeast
40	Late acute PJI	No	Negative	Negative	-	-	No bacteria or yeast
41	Late acute PJI	No	Negative	Negative	-	-	No bacteria or yeast
42	Late acute PJI	Yes, PJI	Negative *	*S. mutans*	*S. mutans (5/6)*	-	No bacteria or yeast
43	Late acute PJI	No	Negative	Negative	Negative	-	No bacteria or yeast
44	Late acute PJI	Yes, PJI	Negative*	Negative	Negative	*S. lugdunensis*	No bacteria or yeast
45	Late acute PJI	Yes, PJI	Negative **, ***	*Ca. jejuni*	*Ca. jejuni (6/6)*	Negative	Gram negative rods

Table S2. Overview of BIOFIRE JI results, Infection Diagnosis, culturing results and Gram staining for all included patients (n=45) with a clinical suspicion of native septic arthritis, early acute PJI and late acute PJI. PJIs were diagnosed according to the 2013 MSIS criteria, septic arthritis according to a positive synovial fluid or tissue culture. * False negative in comparison to infection diagnosis and culturing while the species (marked yellow) is included in the BIOFIRE JI Panel. ** False negative in comparison to infection diagnosis and culturing, but species (marked in red) is not included in BIOFIRE JI Panel. *** False negative in comparison to Gram staining, but species (marked in red) is not included in BIOFIRE JI Panel. **** False negative Gram staining in comparison to BIOFIRE result.

C. = *Corynebacterium*; Ca. = *Campylobacter*; Cu. = *Cutibacterium*; E. = *Enterobacter*; En. = *Enterococcus*; Es. = *Escherichia*; H. = *Haemophilus*; PJI = prosthetic joint infection; SA = septic arthritis; St. = *Staphylococcus*; S. = *Streptococcus*.
